# Effects of Spring Temperatures on the Strength of Selection on Timing of Reproduction in a Long-Distance Migratory Bird

**DOI:** 10.1371/journal.pbio.1002120

**Published:** 2015-04-07

**Authors:** Marcel E. Visser, Phillip Gienapp, Arild Husby, Michael Morrisey, Iván de la Hera, Francisco Pulido, Christiaan Both

**Affiliations:** 1 Department of Animal Ecology, Netherlands Institute of Ecology (NIOO-KNAW), Wageningen, The Netherlands; 2 Centre for Biodiversity Dynamics, Department of Biology, Norwegian University of Science and Technology, Trondheim, Norway; 3 Department of Biosciences, University of Helsinki, Helsinki, Finland; 4 Dyers Brae House, School of Biology, University of St Andrews, St Andrews, Fife, United Kingdom; 5 Department of Zoology and Animal Cell Biology, Universidad del País Vasco (UPV/EHU), Vitoria-Gasteiz, Spain; 6 Department of Zoology and Physical Anthropology, Complutense University of Madrid, Madrid, Spain; 7 Animal Ecology Group, Center for Ecological and Evolutionary Studies, University of Groningen, Groningen, The Netherlands; University College London, UNITED KINGDOM

## Abstract

Climate change has differentially affected the timing of seasonal events for interacting trophic levels, and this has often led to increased selection on seasonal timing. Yet, the environmental variables driving this selection have rarely been identified, limiting our ability to predict future ecological impacts of climate change. Using a dataset spanning 31 years from a natural population of pied flycatchers (*Ficedula hypoleuca*), we show that directional selection on timing of reproduction intensified in the first two decades (1980–2000) but weakened during the last decade (2001–2010). Against expectation, this pattern could not be explained by the temporal variation in the phenological mismatch with food abundance. We therefore explored an alternative hypothesis that selection on timing was affected by conditions individuals experience when arriving in spring at the breeding grounds: arriving early in cold conditions may reduce survival. First, we show that in female recruits, spring arrival date in the first breeding year correlates positively with hatch date; hence, early-hatched individuals experience colder conditions at arrival than late-hatched individuals. Second, we show that when temperatures at arrival in the recruitment year were high, early-hatched young had a higher recruitment probability than when temperatures were low. We interpret this as a potential cost of arriving early in colder years, and climate warming may have reduced this cost. We thus show that higher temperatures in the arrival year of recruits were associated with stronger selection for early reproduction in the years these birds were born. As arrival temperatures in the beginning of the study increased, but recently declined again, directional selection on timing of reproduction showed a nonlinear change. We demonstrate that environmental conditions with a lag of up to two years can alter selection on phenological traits in natural populations, something that has important implications for our understanding of how climate can alter patterns of selection in natural populations.

## Abstract 2

A 31-year study of pied flycatchers shows that it is the temperature at arrival when the offspring return to breed up to two years later that drives selection on breeding time.

## Author Summary

Pied flycatchers are long-distance migrant birds that have advanced their timing of reproduction over the past decades in response to climate change. We studied selection on egg-laying date using a 31-year-long population study and found that in the first 20 years, early-reproducing birds had increasingly higher fitness than late-reproducing birds, resulting in intensified selection on egg-laying date. However, during the last decade, selection on egg-laying date has weakened considerably, although the timing mismatch between breeding and food availability—supposedly the main determinant of selection on breeding phenology—did not change. Whereas conditions during breeding cannot explain the temporal pattern in the strength of selection, spring temperatures at the time the offspring’s first return to the breeding site, i.e., one or two years later, do explain the annual variation in selection well. If spring temperature at arrival is high, early-hatched birds recruit to the breeding population better than late-hatched birds, while in cold years, early- and late-hatched birds recruit equally well. Because early-hatched daughters arrive early when they recruit into the population, females that lay eggs early (and thus have early-hatched chicks) have a strong selective advantage when the following years are warm. Arrival temperatures increased over the first two decades but then cooled again, leading to the observed pattern in selection.

## Introduction

Global climate change has led to shifts in phenology, i.e., the seasonal timing of life history events, such as budburst, flowering, hibernation, migration, or reproduction, in many species. Over the past three decades, plants and animals have shifted their timing, on average, three to four days per decade [1–8]. These shifts allowed them to at least partly adapt to the shifted optimal timing caused by the altered abiotic and biotic conditions. The observed shifts in seasonal timing have often not been as strong as the shift in the optimal timing, which could have negative consequences for fitness [9,10]. In seasonal habitats, reproductive success often increases with the temporal match at the peak of food availability [8,11–15]. Climate change can disrupt this phenological match between trophic levels because of differential phenological responses at different trophic levels [2,8,16], but variation between specific systems is likely depending on the ecology of the interrelated trophic levels [17–21]. In migrant species, these differential responses could be caused by differences in the rates of temperature change in different geographical regions during the annual cycle [22]. Migratory species in particular must rely on environmental conditions experienced en route to predict the optimal time for stopover, arrival, or breeding at distant areas [23,24].

When in a population the timing of life cycle events shifts at a different rate than the optimal timing is shifting, directional selection on the timing will increase. Identifying the environmental variables that are the selective agents is important to predict future impacts of climate change and to be able to study the mechanisms underlying the ecological effects of climate change. However, these selective agents have rarely been identified, even outside the climate change setting, as pointed out earlier [25,26]. There are only a handful of studies that have identified such environmental variables [9,27–30]. One example is selection on timing of reproduction in great tits (*Parus major*) in the Netherlands, where increasing spring temperatures have advanced the peak date in the food for the nestlings (i.e., caterpillars) more strongly than the egg-laying date of the birds [7] (but see [28] for a United Kingdom great tit population where there has been no increase in phenological mismatch). As this mismatch between nestling food availability and demand affects the strength of selection on egg-laying date [9,12,27,28], selection for earlier laying has increased [7]. As the timing of both the caterpillars and the birds depends on temperature, the strength of selection depended on spring temperature [9,27]. Here, we aim to identify the possible selective agents, including phenological mismatch, underlying the selection for egg-laying date in an insectivorous songbird, the pied flycatcher (*Ficedula hypoleuca*).

Pied flycatchers are long-distance migrant birds whose timing of reproduction is constrained by spring arrival time [31]. As for many long-distance migrants, spring arrival dates are advancing slowly, possibly because the environmental cues in the wintering grounds have limited reliability to forecast environmental conditions in the breeding areas [31] (but see [23]). Pied flycatchers in our Dutch long-term study population have advanced their egg-laying date, but not sufficiently enough to keep track of the advancing caterpillar peak [32], and selection for early breeding increased between 1980–1998 [31]. Pied flycatchers breeding in areas with early caterpillar phenology declined strongly, in contrast to areas with later phenology [33], suggesting that the mismatch with caterpillar availability could be determining this change in selection patterns (“phenological mismatch hypothesis” [7,34,35]). Despite these circumstantial data, the environmental variables causing the increased selection for earlier breeding have not been formally tested.

We here aim to (1) describe ongoing changes in selection for timing of reproduction in our long-term pied flycatcher population, spanning more than three decades, using a novel statistical framework, and (2) identify the environmental variables that account for the temporal pattern of selection on timing of reproduction.

To explore the environmental variables potentially underlying selection that could explain this temporal pattern, we analysed not only our primary fitness measure, the number of recruits produced, but also its two components: the number of fledglings produced and the probability that a fledgling recruits as a breeding bird. These two components could be affected by different environmental variables, and therefore analysing them separately may provide further insights in how environmental variables affect the number of recruits produced. As a way to identify the determinants of observed variation in (directional) selection, we assessed the effects of a number of environmental variables (see below) on the relationships between these fitness components and timing of reproduction.

Three types of environmental variables were tested: climatic variables during the breeding season, food availability during the breeding season, and the conditions at the time the offspring returned to breed for the first time. As climatic variables, we considered seasonal patterns in temperature and the duration of rainfall (which reduces the provisioning time for the parents), both during the chick feeding period and during the period just after fledging, i.e., when offspring become independent, as these may affect fledgling survival from early and late broods differently (e.g., [36]). Next, we tested the hypothesis that the degree of temporal mismatch between caterpillar peak dates (as one of the main food sources for offspring [37]) and flycatcher egg-laying dates is important for explaining among-year variation in selection [35]. Finally, we tested an alternative mechanism to explain the observed variation in the annual strength of selection, based on previous observations that in years with low temperatures during spring arrival, early-arriving individuals are less likely to survive [38]. We investigate whether hatch date of an individual (i.e., the lay date of its mother) is a predictor of spring arrival date when offspring return as a breeder and whether early- and late-hatched individuals’ survival upon arrival may thus be affected differentially by spring temperatures.

## Results

**Fig 1:**
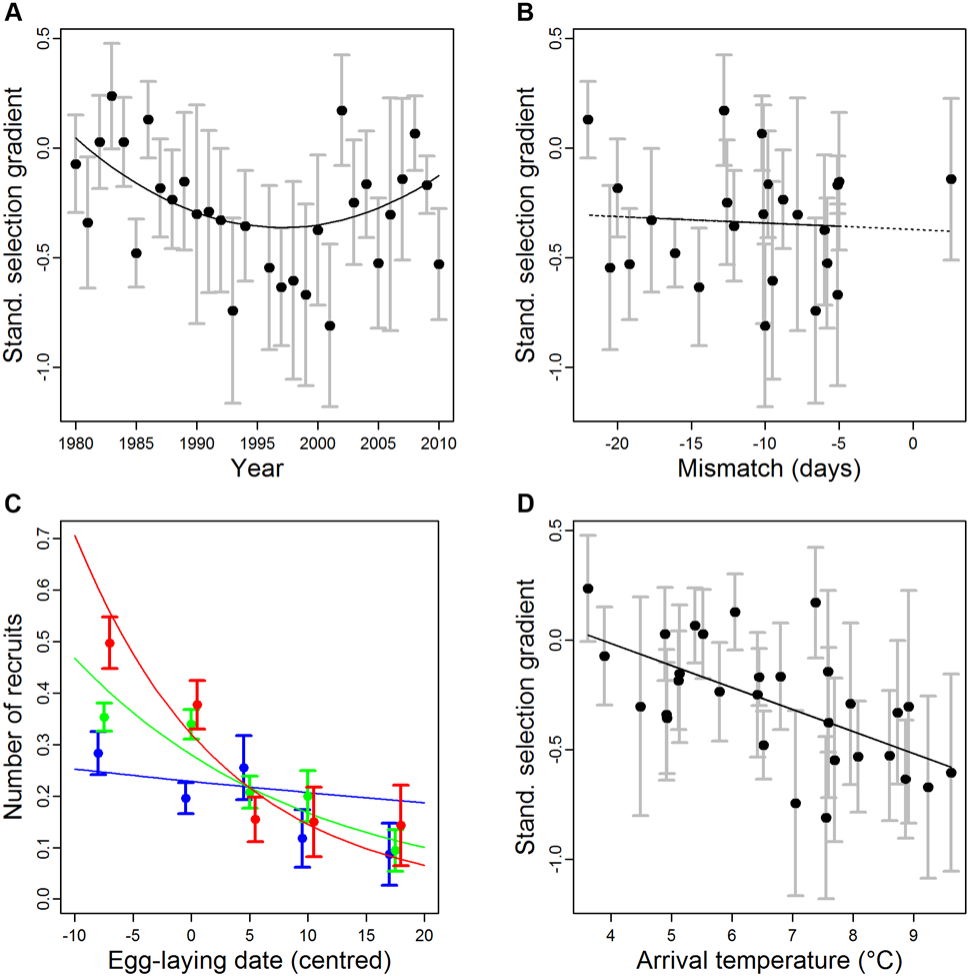
Patterns in selection on timing of reproduction in pied flycatchers using the number of local recruits produced as a fitness measure and environmental variables underlying selection. (A) Standardised linear selection gradients against year and (B) against mismatch between the birds’ population annual mean egg-laying date and timing of caterpillar peak abundance. (C) The relationship between the number of recruits and timing of reproduction (standardised egg-laying date) (see Table 1) becomes steeper with increasing arrival temperatures in the years offspring recruited (note that egg-laying dates and temperatures were grouped in equally sized groups for graphical purposes only; cold spring (<5 C): blue symbols and line, normal spring (5–8 C): green symbols and line, and warm spring (>8 C): red symbols and line). Shown are mean and standard error (s.e.) of number of recruits grouped in 10- to 15-day intervals. Note that this grouping was only done for illustrative purposes. (D) Standardised selection gradients plotted against arrival temperatures. The data used to generate these graphs can be found in S1 Data.

Pied flycatchers have advanced their annual mean egg-laying date between 1980 and 2010 by 12 d (b = -0.38 ± 0.06 d/y, *F*_1,29_ = 40.2, *p* < 0.001). Over the same time period, directional selection in pied flycatchers for earlier egg-laying dates (via numbers of local recruits produced) initially increased in magnitude but waned again in the last ten years of our study period (Fig. 1A; linear and quadratic trend of year in annual linear selection gradients: -0.05 ± 0.04 and 0.12 ± 0.06, respectively, likelihood ratio test (LRT) of model with quadratic trend over model with only linear trend: Chi^2^ = 3.91, df = 1, *p* = 0.048).

**Table 1:** Environmental variables potentially affecting selection on egg-laying date in the pied flycatcher.

	Fitness component					
Environmental variable in interaction with egg-laying date	Number of recruits		Number of fledglings		Recruitment probability	
	Estimate (s.e.)	p	Estimate (s.e.)	p	Estimate (s.e.)	p
*Weather during breeding season*						
Temp_Avg_ (nestling period)	-0.0012 (0.0037)	0.74	0.0023 (0.0013)	0.07	-0.0017 (0.0059)	0.77
RainDuration (nestling period)	0.1376 (0.4367)	0.75	-0.0626 (0.0823)	0.45	0.1642 (0.4210)	0.70
Temp_Avg_ (fledgling period)	0.0006 (0.0061)	0.92	n/a		0.0009 (0.0111)	0.93
RainDuration (fledgling period)	-0.3027 (0.2447)	0.22	n/a		0.2219 (0.2542)	0.38
*Food peak breeding season*						
Pop. mean mismatch (1985–2010)	0.0008 (0.0011)	0.46	-0.0004 (0.0004)	0.33	0.0013 (0.0013)	0.30
Food peak height (1993–2010)	0.0001 (0.0006)	0.87	0.0004 (0.0001)	**0.001**	0.0002 (0.0005)	0.69
*Weather upon return to breed*						
Arrival temperature	-0.0145 (0.0044)	**0.001**	n/a		-0.0141 (0.0049)	**0.004**

We tested the effect of the interaction of the following environmental variables with (centred) egg-laying date on reproductive success: (1) weather variables during the breeding season, calculated for two periods: nestling period and the first weeks after fledging, (2) caterpillar biomass during the breeding season, both the timing relative to the birds’ egg-laying date and the amount of caterpillar biomass (1993–2010 only), and (3) weather variables when the fledglings recruit into the breeding population: the minimum temperatures, averaged for one and two years after fledging, during the females’ “arrival period” (see main text). Estimates from multivariate generalised linear mixed models (GLMMs) with year and female identity as random effects and Poisson or Binomial error distribution and corresponding link-functions. Significant *p*-values are given in bold. The number of fledglings cannot be affected by environmental variables that occur after fledging so these were not tested (n/a in the Table).

Selection has therefore gone from stabilising in the first decade (within-year analysis for the period 1980–1989: linear selection gradient = -0.09 ± 0.07, *p* = 0.18, quadratic selection gradient = -0.24 ± 0.08, *p* = 0.012) to directional selection for earlier egg-laying dates in the second decade (within-year analysis for the period 1990–1999: linear selection gradient = -0.50 ± 0.11, *p* < 0.001; quadratic selection gradient = 0.43 ± 0.20, *p* = 0.02). However, in the last decade, directional selection on egg-laying date has weakened again but without a return to stabilising selection (within-year analysis for the period 2000–2010: linear selection gradient = -0.24 ± 0.06, *p* < 0.0001; quadratic selection gradient = 0.04 ± 0.09, *p* = 0.70).

To explain this pattern in the annual strength of selection on egg-laying date (Fig. 1A), we analysed how the relationships between egg-laying date and the number of recruits produced, and its two components, changed with environmental variables. Surprisingly, the strength of the seasonal decline in the number of recruits was not correlated with the mismatch between egg-laying date and the timing of the seasonal caterpillar peak (interaction egg-laying date * mean population mismatch; Table 1). In accordance with this result, we found no statistically significant relationship between mismatch and standardised linear selection gradients (b = -0.02 ± 0.07, Chi^2^ = 0.094, df = 1, *p* = 0.76, LRT of model including mismatch as well as year and year^2^ versus a model including only year and year^2^, see “Data analysis” in Materials and Methods for details) (Fig. 1B). Thus, the difference between the egg-laying date and the caterpillar peak (the phenological mismatch) does not appear to be a major driver of selection. Additionally, none of the meteorological variables during the breeding season, such as temperature or rainfall duration, explained variation in the strength of the seasonal decline in number of local recruits produced, the number of fledglings produced, or the recruitment probability of fledglings (Table 1). However, the seasonal decline in the number of fledglings produced was stronger when caterpillar peaks were lower, suggesting that the seasonal decline in reproductive output is related to the amount of caterpillars available (interaction egg-laying date * height of the caterpillar peak; Table 1).

We found support for the alternative hypothesis that conditions upon spring arrival for recruiting offspring explain between-year variation in selection on egg-laying date. Thus, when arrival temperatures were warm in the year of recruitment, recruitment probability declined more steeply with the egg-laying date of the clutch the offspring hatched in than when the springs were cold (interaction egg-laying date * arrival temperature; Table 1; Fig. 1C). As pied flycatcher fledglings mostly recruited in the first or second year after fledging (see “Age at recruitment” in Materials and Methods), the arrival temperature was calculated as the mean minimum spring temperature averaged over the two years after hatching during the arrival period of females (see Materials and Methods). In accordance with this, selection for earlier egg-laying date was stronger when arrival temperatures in the two years after fledging were higher (b = -0.18 ± 0.08, Chi^2^ = 4.76, d f = 1, *p* = 0.03; Fig. 1D, LRT of a model including arrival temperature as well as year and year^2^ versus a model including only year and year^2^, see “Data analysis” in Materials and Methods for details), as in these years the early-hatched offspring have higher prospects to be recruited compared to cold years, while the recruitment probability of late-hatched offspring was not affected by arrival temperatures (Fig. 1C). Note that when we fitted either of these year-specific temperatures alone or together (rather than the temperature mean over the two years), we found similar effects (Table 2).

**Table 2:** Separate effects of temperatures during the arrival period one and two year after fledgling on fitness.

	Number of recruits		Recruitment probability	
Arrival temperature	Est. (s.e.)	p	Est. (s.e.)	p
year + 1	-0.0085 (0.0037)	**0.02**	-0.0077 (0.0038)	**0.04**
year + 2	-0.0077 (0.0033)	**0.02**	-0.0085 (0.0035)	**0.02**
year + 1 and year + 2	-0.0074 (0.0036)	**0.04**	-0.0063 (0.0039)	0.10
	-0.0068 (0.0033)	**0.04**	-0.0076 (0.0036)	**0.04**

The interaction between arrival temperature in first and second year and egg-laying date on two fitness components (number of recruits and recruitment probability) was tested using annual temperatures as separate explanatory variables (rather than averaging them, see Table 1). Fledglings return to breed after one or two years, and temperatures in year + 1 and year + 2 after fledging are fitted either on their own (rows 1 and 2) or together (row 3).

**Fig 2:**
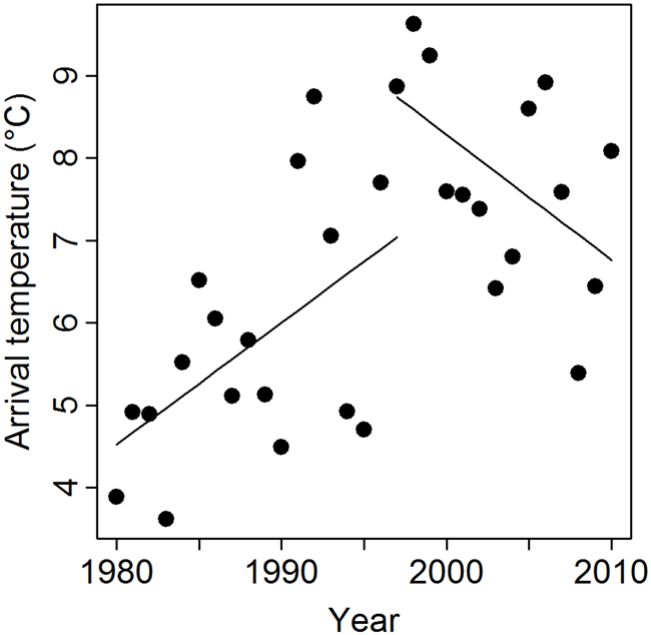
Temporal trend in temperatures during the “arrival period.” Temperatures increased during the first 20 y of the study period and then decreased again (see Results for details on a “broken stick” analysis). The data used to generate these graphs can be found in S2 Data.

To explain the initial increase and later relaxation of selection on egg-laying date (Fig. 1A), arrival temperatures should have changed over time in a similar way, and, indeed, they have: arrival temperatures increased from 4°C to 8°C during the first 20 y of the study period, after which they decreased again over the last decade (year^2^: b = -0.008 ± 0.003, *F*_1,28_ = 9.23, *p* = 0.03; Fig. 2; “broken stick” (or segmented) regression [39] testing for different (linear) trends in different periods: best fit for the two periods 1980–1996 and 1997–2010; in the first period, temperature increased (b = 0.15 ± 0.06, F_1,15_ = 5.44, *p* = 0.03), in the second period temperatures tended to decrease (b = -0.15 ± 0.07, F_1,12_ = 4.45, *p* = 0.057). This temporal pattern in arrival temperatures thus could explain the temporal change in the strength of selection on egg-laying dates (Fig. 1A).

We contend that spring arrival temperatures are important because these temperatures determine ecological conditions affecting survival and settlement just before or after arrival at the breeding grounds. The link with the egg-laying date of a bird’s mother is through a potential effect of hatch date on arrival date later in life. If early-hatched individuals themselves have an early arrival, they may benefit in warmer years but may pay a penalty in colder years (c.f., [38,40]). Indeed, the relative egg-laying date from the clutch in which an individual hatched was positively correlated with the first spring arrival date as adult breeder (relative to the population mean), especially in females (b = 0.22 ± 0.07, F_1,351_ = 8.97, *p* = 0.003), but less so for males (b = 0.11 ± 0.08, F_1,211_ = 1.99, *p* = 0.16). Note that when we constrain the analysis for females to the same observation period as for males (2002–2012), this effect remains statistically significant in females (b = 0.28 ± 0.10, F_1,186_ = 8.02, *p* = 0.005). A further indication that females hatched in early-laid clutches are themselves also arriving and laying eggs early is that egg-laying date is heritable: *h* ^2^ = 0.33 (95% CI: 0.25–0.39).

## Discussion

Between 1980 and 2010, selection on egg-laying date changed from initially stabilising to directional selection for early laying, but this directional selection relaxed again during the last decade. We found no evidence indicating that this pattern was explained by environmental variables measured within the breeding season nor by the temporal mismatch with the food peak [35,41]. Instead, the annual variation in selection was correlated with spring temperatures up to two years after breeding, with stronger selection for earlier laying when spring temperatures in the arrival year for the cohort were high. In our study, the increase in spring temperatures initially led to stronger selection for early egg-laying, as offspring from early nests were more likely to recruit than offspring from late nests under warm conditions upon arrival (Fig. 1C). Between 2000 and 2010, however, arrival temperatures have decreased. This cooling has weakened the strength of directional selection on egg-laying date in this population. We thus show that changes in spring temperatures have a clear impact on selection on a key life history trait: timing of reproduction.

We found no strong evidence for an effect of a phenological mismatch with the caterpillar peak on selection in this migratory species, as previously has been suggested [35,41] based on other systems [9–11,42]. This is unexpected, as synchrony between food peaks and avian timing of reproduction has often been suggested to be important [43,44], and differential phenological responses across trophic levels to climate change have been a major hypothesis explaining increased selection, reduced reproduction, and population declines [45,46]. Also in our study population, caterpillars are an important component in the nestling diet of pied flycatchers, and in warm years, strong seasonal declines in the proportion of caterpillars in the diet were observed [37]. We did find that the height of the peak in caterpillar abundance, rather than the temporal synchrony with the caterpillar peak, affected the seasonal decline in number of fledglings (Table 1). In years with high caterpillar abundance, there was only a weak decline in the number of fledglings over the course of the season; whereas in low caterpillar peak years, this decline was much stronger. This suggests that caterpillars are indeed important as a food source affecting flycatcher fitness and that their availability per se influences selection on seasonal timing. The lack of an effect of synchrony could thus be partly obscured by variation in caterpillar peak height. Caterpillar availability strongly fluctuates between years, with a factor 20 or more between the lowest and highest caterpillar density years [47] and a cycle period of around 9–10 y [48]. Within our dataset, we had periods with peak abundances around 1996 and 2009, and in these years, even birds that breed out of synchrony with the caterpillar peak do not have a strongly reduced offspring production, although very late birds always do poorly because of reduced food availability later in the season [49]. In line with this, the seasonal decline in clutch size is stronger in years when the birds breed late relative to the food peak [50]. Predictions of potential effects of climate change should thus not just consider temporal matching but also potential changes in food abundance [42].

Our fitness measure is the number of fledglings that locally recruit in our population, and therefore this fitness estimate does not include all offspring that survive and disperse out of the study population. Our best estimate is that about four times as many fledglings recruit elsewhere with dispersal distances up to 600 km [51]. This means that measuring fitness as the number of local recruits is potentially biased if differential dispersal exists with respect to laying date of the mother. If offspring that originate from early- or late-laid clutches differ in the likelihood that they recruit outside the study areas, this will lead to apparent selection for egg-laying date. However, correlative and experimental data in pied flycatchers do not support this idea; experimentally delayed hatching date (during three consecutive breeding seasons) did not affect natal dispersal in a metapopulation setup where dispersing offspring could be tracked up to 25 km [52]. Thus, we have no indication that differential dispersal with respect to timing exists in our study system.

The idea of differential dispersal in response to climate change is that late-arriving individuals in warm years benefit by continuing their migration further north and thereby matching habitat phenology with their own timing [53]. Support for northwards dispersal has been found in black-winged stilts in dry springs [54] and in American redstarts that depart later from their wintering grounds [55], but these studies could not unequivocally demonstrate that differential dispersal for a phenotypic trait was related to environmental variation. In our case, we predict that such a pattern would result in especially late-arriving individuals showing high survival in cold years, and low survival in warm years, whereas little effect is expected for early-arriving individuals. Our results, however, do not support these predictions for differential dispersal: late-born offspring recruited equally well when returning in colder or warmer springs (Fig. 1C: comparing data points for laying dates >5). Variation in selection between warm and cold years was thus not caused by differential survival of late-born offspring but rather for early-born offspring that return more in warmer than in colder years (Fig. 1C).

In almost any natural population, permanent dispersal is difficult to separate from mortality, especially when dispersal can occur over large distances. A key consideration in this respect is to assess whether this dispersal likelihood is correlated with the trait of interest, i.e., egg-laying date. We cannot rule out the possibility that differential dispersal affects our fitness estimates, but available evidence suggests that differential dispersal is not a main factor explaining our results. Additionally, differential dispersal would only mediate the patterns observed in Fig. 1A if its relationship with laying date changed over time, which is unlikely. Consequently, in our opinion, the pattern depicted in Fig. 1A is most parsimoniously explained by temporal variation in the relationship between egg-laying date and fitness.

As in many studies of natural populations, the correlation between trait (here egg-laying date) and fitness could potentially be caused by covariance with some environmental variable [56]. Further mechanistic experimental work could therefore be useful in determining whether variation in offspring phenology, and its consequences for offspring and parental fitness, is caused by parental egg-laying date and hence hatch date per se, or if it covaries with parental egg-laying date because of genetic or environmental correlations [57]. We also need more insights into why offspring from early-hatched clutches do so much better when the temperatures are high when they arrive (Fig. 1C). Does an early arrival give them a competitive advantage which is offset against a higher mortality in colder years? This could yield further insights into the evolutionary potential of these phenological traits through parameterisation of more formal models [58,59] of the relationships between parental and offspring phenotype and fitness.

Studies have rarely demonstrated a relationship between selection on life history traits and climate change-altered environmental variables (i.e., selective agents [25]). Yet, all of these studies found immediate effects, i.e., within the same season in which the life history trait is expressed [9,28,29]. Here, we have shown the apparent absence of such immediate effects but demonstrated an effect of conditions years after the life history trait is expressed. Such effects have been little studied in this context, but might be common. Studies of both delayed effects of environmental variables and the interaction between demographic and evolutionary processes on selection are urgently needed as selection is a key component for microevolution of life history traits, which is ultimately the only route for populations to adapt to novel environments [60].

## Materials and Methods

### Ethical Statement

This study was carried out with the approval of the Animal Experimentation Committee of the Royal Dutch Academy of Sciences.

### Study Area and Field Work

We analysed data from 1980 until 2010 from the Hoge Veluwe study area (52°059 N, 05°509 E, the Netherlands), where about 400 nest boxes are supplied in a 171 ha study area of mixed deciduous and coniferous forest. We only analysed data until 2010 to get reliable estimates of the number of recruits produced since about half of the offspring is found to recruit only two years after fledging as a breeding individual (see “Age at recruitment” in Materials and Methods). The broods of 1995 were excluded from all analyses since in this year almost all broods in the population were manipulated in a way that affected their breeding success [61].

Nest boxes were checked weekly during the egg-laying period, and first egg-dates (henceforth called egg-laying dates) were calculated on the assumption that one egg is laid per day. We included only clutches that we considered as first clutches of females: repeats of known females that failed were excluded, as were all clutches that started >30 d after the first egg-laying date in that year. During these regular checks, nest-building stages were reported. Adults were caught during chick feeding using nest box traps for identification based on their ring numbers. All nestlings were ringed with standard aluminium rings at an age of 7 to 12 d.

### Capture Probabilities

The observed temporal pattern in the strength of selection (Fig. 1A) could be caused by a systematic change over the study period in how the adult recapture probabilities differed for birds hatched in late or in early-laid clutches. In order to test this possibility, we estimated survival and capture probabilities using a formal capture-mark-recapture analysis [62] using the software MARK [63]. We first fitted simple models, in which adult survival and recapture probability were constant over years and possibly only differed among the sexes. We then tested whether survival and recapture probabilities would differ among years and whether they depended on the egg-laying date of the clutch an individual was born in (“birth clutch lay date”), and whether this relationship differed between years, by adding the interaction of “birth clutch lay date” with year to the model. If recapture probabilities would depend on “birth clutch lay date,” and this relationship would differ among years, it could possibly bias our results.

To test whether recapture probabilities could explain the observed selection and its temporal change, we fitted a range of capture-mark-recapture models in which recapture probability depended on sex, year (linear and squared effect), and the egg-laying date of the clutch from which an individual hatched. While there was clear support for a linear temporal change in recapture probability (Table 3, model 1), the support for a quadratic change of recapture probability over time was less clear (Table 3, model 2: Δ AICc = 2.0 and AICc weight = 0.27). However, we found no evidence for capture probability being correlated with birth clutch lay date (Table 3, model 4: Δ AICc = 9.7 and AICc weight = 0.005).

**Table 3:** Results of capture-mark-recapture analysis.

Model	AICc	Δ AICc	AICc Weight	Likelihood	No. parameters	Model deviance
1 Φ(s) p(s + y)	2704.5	0	0.71	1.00	5	2694.5
2 Φ(s) p(s + y + y^2^)	2706.5	2.0	0.27	0.37	6	2694.4
3 Φ(s) p(s)	2713.5	9.0	0.008	0.011	4	2705.46
4 Φ(s) p(s + ld)	2714.2	9.7	0.005	0.008	5	2704.20
5 Φ(.) p(.)	2714.5	10.0	0.005	0.007	2	2710.48

Survival probability (Φ) and recapture probability (p) were modelled depending on sex (s), year (y), year squared (y^2^), and egg-laying date of the clutch in which the individual was born (ld).

### Male and Female Arrival Dates

Male arrival date was determined from 2002 to 2010 through daily observations covering the whole study area, from April to early May by one to three trained observers. In our pied flycatcher population, males greatly vary in their plumage characteristics (i.e., size and shape of forehead patch and darkness of dorsal feathers; see [64]), and about half of them had been marked in previous years with aluminium and colour rings. We made notes of these features during our daily observations to characterise males singing at each nest box, allowing ascribing arrival dates—based on the similarity of features—to individual males, which were captured during chick feeding in the nest boxes. Pied flycatcher males commonly breed in the immediate surroundings of the area where they are first observed singing [65], and hence in most cases the description of the male repeatedly singing in a particular nest box matched the characteristics of the bird captured in it during breeding, which supports the validity of this approach to gather information on spring arrival phenology in this species (see [65] for a similar approach). This procedure allowed us to assign the arrival date in 571 cases (397 different individuals) in nine years, which is more than half of the males present in our study site.

For female arrival date, we used the estimated start of nest building as proxy. Female flycatchers select a mate within hours upon their arrival [66,67], and they start nest building immediately after. Over the entire study period, the stage of nest building was scored (using a six-point scale, from little material to a complete nest) during the weekly nest checks. We assumed that little material meant that nest building had started the day of the check, whereas a complete nest started six days (i.e., the day after the previous nest box check) earlier (and other stages in between). The validity of this proxy was tested using observational data on arrival dates of male and female pied flycatchers in a nearby population (Dwingeloo, The Netherlands).

The Dwingeloo study area has 100 nest boxes and approximately 50 pairs of pied flycatchers annually. The same observer (CB) checked arriving pied flycatcher on a mostly daily basis from April 10 to May 15 for 2007, 2008, 2009, 2010, and 2013. Note that the years 2010 and 2013 were exceptionally cold springs, resembling what was normal during the early part of our time series (1980s), and therefore this allows us testing under the full range of environmental circumstances whether this proxy indeed can be used. Male flycatcher arrival was determined based on individual plumage characteristics (see above). Female arrival date was determined as the pairing date, which was often clearly visible because males reduced or completely stopped singing after being paired, and these behavioural changes were mostly confirmed by the observation of a female with the focal male. Nest boxes were checked at least once every five days, but more often to confirm female arrival, and the contents of the box were noted on the same six-point scale as at the Hoge Veluwe.

To test whether female arrival date correlated well with the start of nest building, we used 194 observations in which female arrival date and the start of nest building were known. The estimated slope was 1.05 ± 0.02 (t_1_ = 66.84, *p* < 0.001). These data clearly show the validity of using the start of nest building as proxy for female arrival, as there is an almost 1:1 relationship. However, it must be noted that if females give up their nest and renest, their arrival dates cannot be estimated from the start of nest building. Especially late nests therefore should be treated with caution, adding noise to the data and, especially in cases where a large fraction of nests are abandoned, this proxy becomes inappropriate. In our Hoge Veluwe study population, however, this is rarely the case.

### Fitness Variables

We used the number of offspring produced that return as a breeding bird in the Hoge Veluwe population (local recruit) as a fitness measure. This number of recruits produced is the total number of recruits from first and replacement clutches (after a failed first clutch) within a single season.

The number of recruits produced can be partitioned in two components, the number of fledglings produced and the probability for these fledglings to recruit. These two components can be affected by very different climatic variables, and hence we also analysed how these two components were affected by the selected climatic variables to gain more insight in how our overall fitness measure, the number of recruits, was related to climate variables. The number of fledglings was the number of chicks ringed minus the number of offspring found dead in the nest box after the offspring had fledged. The probability to recruit was calculated as the number of recruits per nest divided by the number of fledglings per nest (thus excluding the nests without fledglings). As capture probability of a locally recruiting offspring was not correlated with the egg-laying date of the brood a recruit originated from (see above and Discussion), we did not correct the number of recruiting offspring for capture probability. Note that an offspring is a recruit when it is caught at any age at the Hoge Veluwe, so even when birds are missed in their first breeding year, they are likely to be caught in their second year of breeding.

### Age at Recruitment

Most locally-hatched pied flycatchers are caught as breeding adults for the first time either in their first or second year after fledging: 32% of all male recruits returned to breed for the first time at age one and 54% at age two; this pattern is almost exactly reversed in female recruits, of which 59% returned at age one and 32% at age two. Only 14% (males) and 9% (females), respectively, returned at older ages to breed for the first time. Given our capture probabilities (see above), the records of birds breeding for the first time two years after fledging is unlikely to be due to birds breeding but not being identified in their first year after fledging.

### Environmental Variables

Data on daily mean and minimum temperature (°C) and rain duration (number of hours of rain in 24 hours) from the weather station directly adjacent to the study area (Deelen, 52°059 N, 05°509 E) were obtained from the Royal Dutch Meteorological Institute (KNMI; http://www.knmi.nl/klimatologie/uurgegevens/#no). We used daily mean temperature as the predictor variable for the nestling period (defined per year as the period from the mean egg-laying date plus 18 d (6 d of egg laying plus 12 d of incubation) to mean egg-laying date plus 30 d (an additional 12 d of chick rearing) and the fledging period (defined per year as the period of the mean egg-laying date plus 31 d to mean egg-laying date plus 42 d (12 d of post-fledging care [64]). We used minimum temperature for the arrival period because we hypothesised that while nestling survival would be affected by a variety of factors better reflected by mean temperature, the survival of recruits upon arrival would depend on the severity of harsh conditions reflected by minimum temperatures.

Temperatures at the time of arrival in spring were calculated during the period when 90% of all females arrived (averaged over the 30 y). This “arrival period” was defined as the interval from the average annual 5% quantile (between-year standard deviation 5.3 d) until the average annual 95% quantile (between-year standard deviation 6.7 d) of female arrival dates (using nest building as proxy): April 23 until May 12. As most pied flycatchers were caught breeding for the first time either in their first or second year after fledging (see “Age at recruitment” in Materials and Methods), we took the average daily minimum temperature during the arrival period one and two years after fledging as a measure of environmental conditions upon first arrival. We used temperatures averaged over a fixed period because using temperatures measured over the realised arrival period, i.e., an annually variable period, would “reverse” causality: in that case, the behaviour of the birds would have determined the climatic variable rather than vice versa.

Caterpillars form an important prey for the nestlings of many passerine bird species including pied flycatchers [37], and caterpillar biomass has been monitored between 1985–2010 in the Hoge Veluwe-study area by collecting caterpillar droppings (“frass”) using special nets placed on the ground under oak trees (*Quercus robur*). “Frass” was collected two to three times a week, dried, sorted, and weighed. Caterpillar biomass was then calculated from its weight using the formula given in [68]. See [11] for more details on study area and the described methods.

### Data Analysis

We estimated annual standardised directional selection gradients following methods outlined in [69]. Briefly, we first characterised the relationship between egg-laying date and the number of local recruits produced, in each year using generalised linear models with a Poisson error structure. GLMs included linear and quadratic linear predictor scale regression terms (to detect directional, stabilising or disruptive selection), except for 1981, 1989, 1990, 1991, and 2006, where we fitted only linear regression terms because low numbers of recruits (< 15) precluded fitting more complex models. Next, we used the R package gsg [69] to obtain yearly standardised directional selection gradients (sensu [70]), and their standard errors and *p*-values using a parametric bootstrap algorithm. Because of the large statistical uncertainty associated with each annual selection gradient, we developed a method to robustly estimate the regression of selection gradients against predictors (e.g., year or environmental variables). We fitted the model β_*t*_ ~ μ + *b*_1*t*_ + *b*_2*t*_^2^ + *m*_*t*_ + *e*_*t*_, where β_*t*_ are estimated selection gradients for year t, and *m*_*t*_ are deviates of estimated selection gradients from their unknown true values and are assumed to be drawn from the distributions N(0, SE_*t*_^2^), where SE_*t*_ are the standard errors associated with each estimated selection gradients. *b*_1_ and *b*_2_ are regression coefficients relating selection to year. When testing whether other environmental variables affected selection strength, coefficients relating selection to these environmental variables were included similarly in addition to linear and quadratic effects of year. *e*_*t*_ are residuals, and are assumed to be normally distributed with estimated variance. We fitted the model by maximum likelihood, tested regression terms with LRTs, and approximated the standard errors of regression coefficients from the information matrix. The maximum likelihood techniques provided nearly identical inferences to a complimentary Bayesian approach implemented by extending the meta-analytic approaches used in [71]. When formally analysing stabilising selection, we pooled data (for reasons mentioned above), into the periods (1980–1989, 1990–1999, and 2000–2010) and regressed relative fitness against the linear and quadratic term of centred egg-laying date using the approach described above.

The relationship of single fitness components (number of recruits, number of fledglings and recruitment probability) with egg-laying date and environmental variables was tested with GLMMs, with year as random effect, to account for nonindependence of data from the same year with respect to the environmental variables that were measured at an annual scale. Female identity was included as random effect to account for females breeding in more than one year. Number of fledglings and recruits were analysed using a log-link and a Poisson error-distribution. An observation-level random effect was included in the Poisson models, the commonly observed overdispersion in reproductive success [72]. The recruitment probability per brood was analysed using a logit-link and a Binomial error-distribution.

The heritability of egg-laying date was calculated based on standard quantitative genetic approaches implemented in the so-called “animal model” ([73]. To account for year-to-year variation and age effects, year and age were included as fixed factors. Female identity was included as “permanent environment” random effect to account for repeated breeding events of the same female. The additive genetic effect was included as random effect to estimate heritability as the ration of the variance explained by the additive genetic effect over the total phenotypic variance (excluding year and age effects as these were fitted as fixed effects).

All estimates are reported ± standard error.

Data deposited in the Dryad repository: http://dx.doi.org/10.5061/dryad.cv24c [74].

## Supporting Information

S1 Data The data used to generate Fig. 1. (XLSX)

S2 Data The data used to generate Fig. 2. (XLSX)

## Abbreviations

LRT: likelihood ratio test
GLMM: generalised linear mixed model
s.e.: standard error
